# Slit-Robo GTPase-Activating Protein 2 as a metastasis suppressor in osteosarcoma

**DOI:** 10.1038/srep39059

**Published:** 2016-12-14

**Authors:** Tracy A. Marko, Ghaidan A. Shamsan, Elizabeth N. Edwards, Paige E. Hazelton, Susan K. Rathe, Ingrid Cornax, Paula R. Overn, Jyotika Varshney, Brandon J. Diessner, Branden S. Moriarity, M. Gerard O’Sullivan, David J. Odde, David A. Largaespada

**Affiliations:** 1 University of Minnesota, Masonic Cancer Center Minneapolis, MN, USA; 2 Department of Biomedical Engineering University of Minnesota, Minneapolis, MN, USA; 3 Comparative Pathology Shared Resource, University of Minnesota, Minneapolis, MN, USA; 4 Department of Pediatrics, University of Minnesota, Minneapolis, MN, USA; 5 Center for Genome Engineering, University of Minnesota, Minneapolis, MN, USA; 6 College of Veterinary Medicine, Department of Veterinary Population Medicine, University of Minnesota, Minneapolis, MN, USA

## Abstract

Osteosarcoma is the most common primary bone tumor, with metastatic disease responsible for most treatment failure and patient death. A forward genetic screen utilizing *Sleeping Beauty* mutagenesis in mice previously identified potential genetic drivers of osteosarcoma metastasis, including Slit-Robo GTPase-Activating Protein 2 (*Srgap2*). This study evaluates the potential role of SRGAP2 in metastases-associated properties of osteosarcoma cell lines through *Srgap2* knockout via the CRISPR/Cas9 nuclease system and conditional overexpression in the murine osteosarcoma cell lines K12 and K7M2. Proliferation, migration, and anchorage independent growth were evaluated. RNA sequencing and immunohistochemistry of human osteosarcoma tissue samples were used to further evaluate the potential role of the Slit-Robo pathway in osteosarcoma. The effects of *Srgap2* expression modulation in the murine OS cell lines support the hypothesis that SRGAP2 may have a role as a suppressor of metastases in osteosarcoma. Additionally, *SRGAP2* and other genes in the Slit-Robo pathway have altered transcript levels in a subset of mouse and human osteosarcoma, and SRGAP2 protein expression is reduced or absent in a subset of primary tumor samples. SRGAP2 and other axon guidance proteins likely play a role in osteosarcoma metastasis, with loss of SRGAP2 potentially contributing to a more aggressive phenotype.

Malignant bone tumors comprise the eighth most common type of pediatric cancer in the United States, with the majority being osteosarcoma^1^,^2^. Osteosarcoma has a bimodal age distribution, with the first and primary peak incidence in adolescence and a second smaller peak incidence starting in the sixth decade of life^2^. Osteosarcoma survival dramatically improved with the introduction of high-dose chemotherapy into treatment regiments in the 1970’s^3^. However, the 5-year survival rate has remained around 60% for the past four decades despite advanced surgical techniques and numerous clinical trials^3^,^4^. Metastatic disease, which typically occurs in the lungs, is the main cause of treatment failure and patient death. Targeted therapeutics are needed to improve patient survival beyond what can be offered by surgery and non-specific chemotherapy.

Osteosarcoma has a high tendency for aneuploidy, chromothripsis, and kataegis, all indicative of chromosomal instability^5^,^6^,^7^. Given the complex chromosomal changes acquired through genomic instability, identifying specific genes involved in osteosarcoma tumorigenesis and progression is challenging. Little overlap between potential drivers of osteosarcoma exists between genetic studies of primary tumors owing to the complexity and the heterogeneity in osteosarcoma. To address this, a forward genetic screen of osteosarcoma utilizing the *Sleeping Beauty* mutagenesis system was previously conducted to identify potential drivers of osteosarcoma development and metastasis^8^.

The *Sleeping Beauty* mutagenesis screen identified 232 sites associated with osteosarcoma development and 43 sites associated with metastasis. Slit-Robo GTPase-Activating Protein 2 (*Srgap2*) was the most frequently mutated gene in metastatic nodules after *Pten (Srgap2*: n = 6/19 animals with metastasis, 31%). Primary tumors were evaluated from mice with a *Trp53* deficient background in addition to transposon mutagenesis and from mice with transposon mutagenesis alone. *Srgap2* had the fifth most insertions in primary tumors (n = 13/96, 13.5%) within the subgroup of mice on a *Trp53* deficient background in addition to transposon mutagenesis.

The physiological roles of SRGAP2 have primarily been studied in the context of cortical neuron function^9^,^10^,^11^,^12^. SRGAP2 has three domains: an SH3 domain by which it binds Robo proteins embedded in cellular membranes^13^, an F-BAR domain through which it induces filopodia formation^9^,^11^,^12^, and a Rho-GAP domain that activates the GTPase of the F-actin modulating Rho-GAP protein Rac1^9^,^14^. *SRGAP2* underwent 3 duplication events throughout evolution in the human species, creating *SRGAP2B, SRGAP2C,* and *SRGAP2D*, which are truncated versions of the full-length gene sharing 99% sequence similarity^15^. SRGAP2B and SRGAP2C have been shown to act as dominant negative inhibitors of SRGAP2 in laboratory experiments^10^; however, *SRGAP2B* is predicted to produce mostly isoforms with premature stop codons, rendering the protein product non-functional^15^. *SRGAP2D* also has a premature stop codon and its product is predicted to undergo nonsense mediated decay^15^. In population studies, *SRGAP2* and *SRGAP2C* have a fixed diploid copy number, whereas *SRGAP2B* and *SRGAP2D* vary from 0 to 4 copies, which is further evidence of the more critical physiological role of SRGAP2 and SRGAP2C compared to SRGAP2B and SRGAP2D^15^.

Knockdown of *SRGAP2* or expression of the dominant negative SRGAP2C has revealed an increase in neuron migration through mouse brain slices electroporated *ex vivo*, whereas over-expression of SRGAP2 encourages neurite outgrowth and branching, thereby decreasing neuron migration^9^,^10^. SRGAP2 has been shown to induce similar phenotypes in the human colon cancer HCT116 cell line *in vitro*^16^, supporting the potential role of acting as a suppressor of migration in the context of cancer. Here we functionally evaluate the potential role of SRGAP2 in osteosarcoma development and metastasis.

## Results

### Sleeping Beauty forward genetic screen implicates Srgap2 as a tumor and metastasis suppressor gene

*Srgap2* was recurrently mutated in four *Sleeping Beauty* screens documented in the Candidate Cancer Gene Database, a database of cancer driver genes from *Sleeping Beauty* transposon based forward genetic screens in mice^17^. Our prior osteosarcoma^8^ and malignant peripheral nerve sheath tumor^18^ screens predicted a disrupted gene function (Supplementary Table S1). The chronic myeloid leukemia^19^and medulloblastoma^20^ screens did not evaluate the predicted effect on gene function. The percent of primary tumors with an insertion in *Srgap2* ranged from 13.5% in osteosarcoma to 30% in chronic myeloid leukemia (Supplementary Table S1).

**Figure 1:**
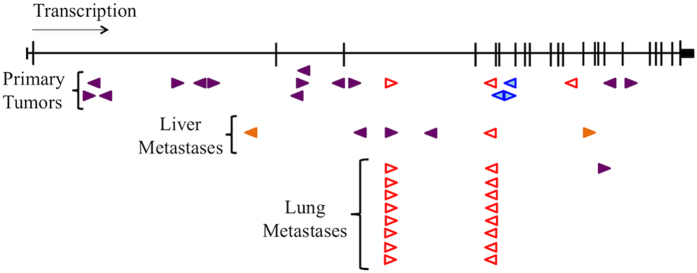
*Sleeping Beauty* transposon insertion profile predicts Srgap2 is a tumor suppressor. Arrows denote transposon insertion sites, which point in the direction of the promoter. Three animals had multiple insertions in *Srgap2*, represented by the red open arrows, the blue shaded arrows, and the solid orange arrows. All remaining insertion sites, represented by solid purple arrows belong to different animals. The insertion profile predicts Srgap2 is a tumor suppressor due to the unbiased transposon insertion throughout the gene without a promoter direction preference.

The mutagenic transposons used in the *Sleeping Beauty* screens can disrupt gene transcription in either orientation, but can only promote gene transcription in one direction^8^. The insertion locations and the promoter direction of transposons within *Srgap2* in the primary osteosarcoma tumors and metastatic nodules reveal a profile indicative of a tumor suppressor gene: the transposons are inserted throughout the gene without a bias in promoter orientation (Fig. 1). Insertions were found in 15 primary tumors from mice with and without a *Trp53* deficient background in addition to transposon mutagenesis (n = 119). RNA sequencing was performed on most primary tumor samples from the osteosarcoma screen (n = 105/119) and mapped using a modified genome containing a *Sleeping Beauty* chromosome with the T2/Onc sequence^21^. Ten samples were found to contain reads in *Srgap2* paired to reads in the transposon sequence. Evaluation of the mapping patterns around the insertion sites was used to determine the impact of the transposon insertion on *Srgap2* expression. Of the 10 samples, 7 appeared to result in a substantial reduction in normal *Srgap2* expression (Supplementary Table S2). These data suggest that loss of *Srgap2* may be involved in tumor formation in a subset of *Sleeping Beauty* induced murine osteosarcoma. To functionally evaluate the potential role of Srgap2 in osteosarcoma development and metastasis, we overexpressed and knocked out *Srgap2* in murine osteosarcoma cell lines.

### Overexpression and knockout of SRGAP2 in murine osteosarcoma cell lines

The murine genome only contains the mouse ortholog of the ancestral *SRGAP2* gene, which has a 98% amino acid similarity to human *SRGAP2.* Expression of the human ancestral gene has been shown to induce consistent phenotypes in murine cells^10^. The murine osteosarcoma cell lines K12, created from a spontaneous tumor, and a highly metastatic derivative called K7M2, created through *in vivo* passaging, were used in this study^22^ *SRGAP2* overexpression was achieved with doxycycline inducible *PiggyBac* vectors containing human *SRGAP2* cDNA. *Srgap2* knockout was accomplished using the CRISPR/Cas9 system.

**Figure 2:**
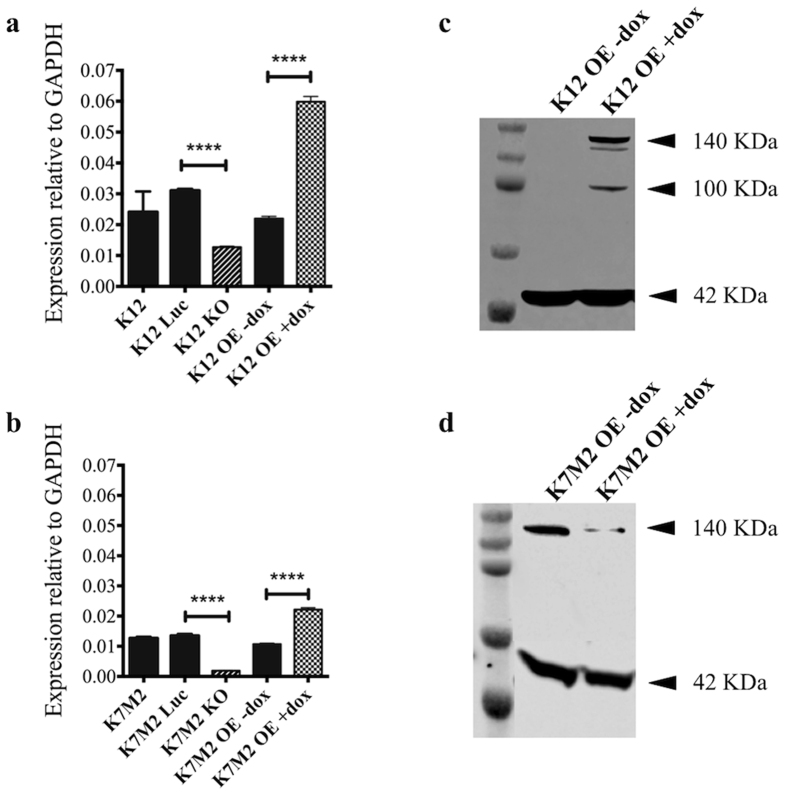
*SRGAP2* mRNA xpression levels in K12 and K7M2 cell lines by quantitative RT-PCR and protein quantification by Western blotting. (**a,b**) *SRGAP2* mRNA levels were quantified by RT-PCR. The expression of *SRGAP2* mRNA is >2 fold overexpressed in cell lines with the addition of doxycycline. Expression of *Srgap2* mRNA is (**a**) halved in the K12 KO and (**b**) not detectable in the K7M2 KO. Bar graph shows mean ± SEM (n = 3). (**c,d**) Western blotting showing the addition of doxycycline to culture media induces expression of human SRGAP2 protein in the (**c**) K12 and (**d**) K7M2 OE cell lines. The antibody does not bind endogenous murine SRGAP2 protein. Four bands are observed: SRGAP2 (140 KDa), beta-actin control (42 KDa), and a two unknown bands (120 KDa and 100 KDa). ****p-value < 0.0001 (two-tailed, unpaired t-test).

The K12 knockout (KO) cell line has half the expression of *Srgap2* mRNA compared to parent and luciferase controls (qPCR) (Fig. 2a). Genomic sequencing of the targeted exon in the K12 KO line showed deletions and insertions in half of the sequencing samples (Supplementary Fig. 1). The K7M2 KO has no detectable expression of *Srgap2* mRNA compared the parent and luciferase controls (qPCR) (Fig. 2b), and genomic sequencing showed deletions and insertions in all of the samples (Supplementary Fig. 1). This data suggest that K12 most likely has heterozygous knockout of *Srgap2*, whereas K7M2 has homozygous knockout of *Srgap2.*

Overexpression of human *SRGAP2* cDNA was accomplished by the addition of doxycycline to cell lines stably expressing a tetracycline-responsive promoter driving the expression of *SRGAP2*. The level of *SRGAP2* mRNA expression in the K12 and K7M2 overexpression (OE) cell lines exposed to doxycycline is at least doubled by qPCR compared to overexpression lines without doxycycline (Fig. 2a,b). Expression of the human *SRGAP2* transgene in the K12 OE and K7M2 OE cell lines was further evaluated by Western blotting (Fig. 2c,d). Numerous commercial antibodies were tried against the murine lysates, but none could detect endogenous murine SRGAP2 in wild type lysates, as demonstrated by the OE –dox lysates in Fig. 2b. Western blotting could, therefore, not be used to assess *Srgap2* knockout in the murine cell lines.

Four bands were observed in the K12 OE cell line and three bands in the K7M2 OE cell line with induced human *SRGAP2* expression (140 KDa: SRGAP2, 120 KDa: unknown, 100 KDa in K12 OE: unknown, 42 KDa: beta-actin). SRGAP2 has a predicted molecular weight of 121 KDa, but most antibodies, including the Abcam antibody (#ab121977) used here, detect a band at 140 KDa. A separate group working on SRGAP2 noted a second band of slightly lower molecular weight on Western blotting that was described as non-specific binding^23^. A similar band was observed around 120 KDa in this work when doxycycline was administered to induce expression of the human *SRGAP2* transgene. It is possible that the two bands represent different products of the *SRGAP2* gene, with the 140 KDA band representing a post-translationally modified form of SRGAP2. Another unknown band around 100 KDa was also observed in the K12 murine OE cell line. The appearance of this band when the cells are treated with doxycycline to induce expression of the human SRGAP2 protein suggests there may be an alternative in-frame ATG start codon or that it is a degradation product of SRGAP2.

### SRGAP2 expression affects *in vitro* proliferation and migration but not anchorage independent growth in murine osteosarcoma cell lines

**Figure 3:**
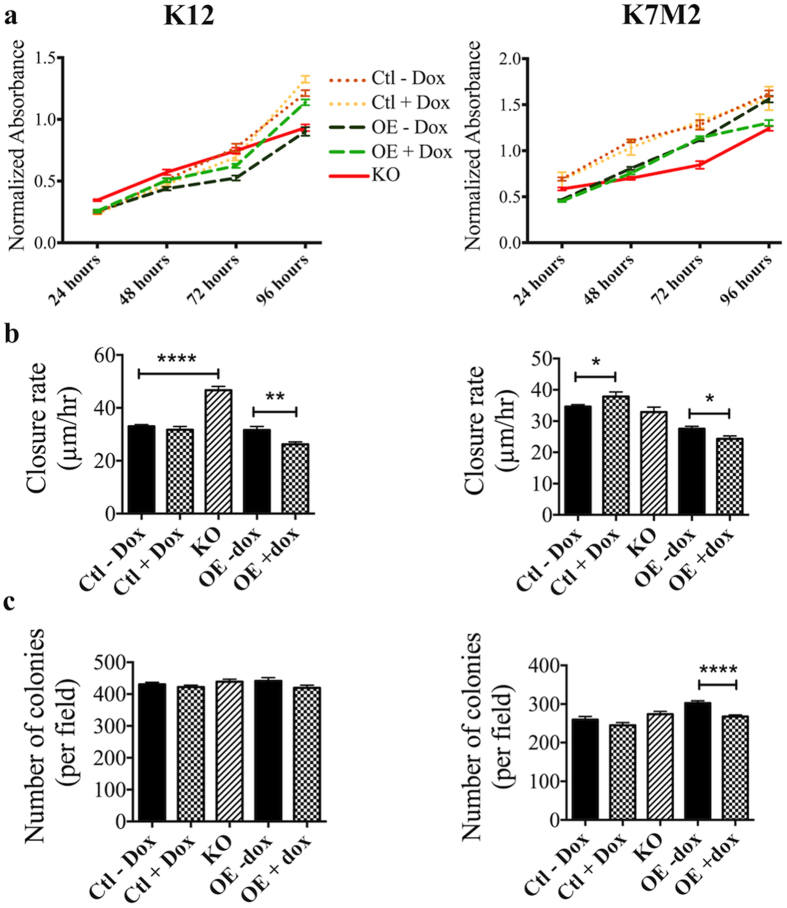
*SRGAP2* expression affects *in vitro* proliferation and migration but not anchorage independent growth in murine cell lines. (**a**) MTS assay was used to evaluate cellular proliferation. Readings were taken at 24, 48, 72, and 96 hours of incubation. A slowed cellular proliferation was observed in K12 and K7M2 KO cell lines. Each point shows mean ± SEM (n = 18). (**b**) Wound-healing assay was used to evaluate cellular migration. Overexpression of *SRGAP2* suppressed cellular migration in both K12 and K7M2 cell lines. Knockout of *Srgap2* enhanced cellular migration in K12 but not K7M2 cell lines. Bar graphs show mean ± SEM (n ≥ 16). (**c**) Soft agar colony formation assay was used to evaluate anchorage independent growth. *SRGAP2* expression did not influence anchorage independent growth of K12 cell lines, whereas *SRGAP2* overexpression decreased the K7M2 cell line’s ability for anchorage independent growth. Bar graphs show mean ± SEM (n = 36). *p-value < 0.05, **p-value < 0.01 ****p-value < 0.0001 (two-tailed, unpaired t-test).

Cell densities and rates of proliferation for the K12 and K7M2 *Srgap2* KO, *SRGAP2* OE, and control cell lines were compared at 24, 48, 72, and 96 hours post plating. K12 parent and K7M2 luciferase with and without doxycycline are shown as the control lines. The luciferase and parent cell lines behaved similarly in all assays. A p-value of statistical difference was found by ANOVA at all time points, indicating variance among the K12 and the K7M2 cell lines (Supplementary Table S3). The addition of doxycycline to induce *SRGAP2* expression in the OE cell lines did not substantially affect proliferation (Fig. 3a). The K7M2 KO showed significantly less proliferation compared to control at all time points (p < 0.0001). The K7M2 KO grew slower at the early time points, but the proliferation rate increased between 72 and 96 hours (fold change, Supplementary Table S3). The K12 KO showed significantly less proliferation compared to control at 96 hours (p < 0.0001). However, the K12 KO had a linear growth rate and the fold change in absorbance was lower than the control cell lines at all time points (fold change, Supplementary Table S3). These data suggest that SRGAP2 may reduce cellular proliferation.

Enforced *SRGAP2* expression at high levels slowed the migration of both K12 (p = 0.001) and K7M2 (p = 0.02) OE cell lines in wound healing assays (Fig. 3b). Although a difference was also observed in the K7M2 control with the addition doxycycline (p = 0.04), the phenotypic effect was opposite that of the OE line with doxycycline. The presence of doxycycline likely did not directly contribute to the observed difference in migration. Knockout of *Srgap2* increased cellular migration of the less aggressive K12 cell line (p < 0.0001) but did not have an effect on migration in the K7M2 cell line. Anchorage independent cell growth was suppressed when *SRGAP2* expression was induced in the K7M2 OE cell line (p < 0.0001) (Fig. 3c). All other K12 and K7M2 cell lines had similar performance in anchorage independent growth.

### Expression of SRGAP2 and other genes in the Slit-Robo pathway are altered in human osteosarcoma samples

**Figure 4:**
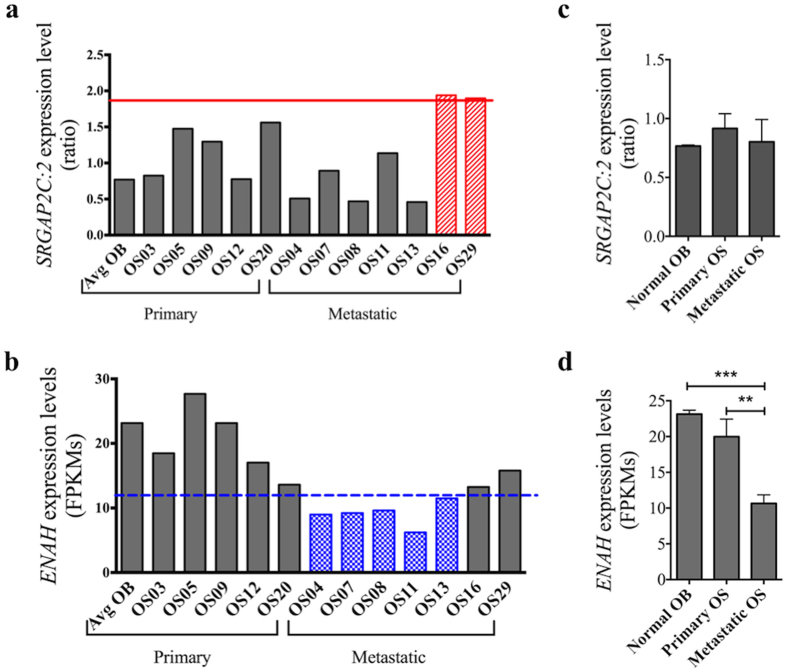
RNA expression changes in *SRGAP2C*: *SRGAP2* ratio and *ENAH* observed in human metastatic osteosarcoma samples compared to primary osteosarcoma tumor samples. In a small cohort of osteosarcoma samples, 5 taken from primary tumors and 7 taken from metastatic tumors, the metastatic samples either showed (**a**) a greater than 1.8 fold increase in the *SRGAP2C*: *SRGAP2* ratio (n = 2) or (**b**) a greater than 2 fold decrease in *ENAH* expression (n = 5). On average, (**c**) the *SRGAP2C: SRGAP2* expression ratio was similar across all groups, but (**d**) the level of *ENAH* expression was significantly reduced compared the osteoblasts (OB) and primary osteosarcoma (OS) samples. Naming convention for the samples provided in Supplementary Table S6. Bar graphs in panel (**c**,**d**) show mean ± SEM. **p-value < 0.01 ***p-value < 0.001 (two-tailed, unpaired t-test).

To evaluate SRGAP2’s role in human osteosarcoma, mRNA expression levels of genes associated with the Slit-Robo pathway were evaluated in 12 juvenile osteosarcoma samples for which RNA sequencing data were available (Supplementary Table S5)^7^. The list of genes was compiled from pathway information for SRGAP2 provided by Gene Cards (www.genecards.org). Metastatic samples had either 1.8 greater *SRGAP2C*: *SRGAP2* transcript ratio (n = 2) (Fig. 4a) or a two-fold reduction of *ENAH* compared to primary osteosarcoma samples (n = 5) (Fig. 4b). On average the *SRGAP2C: SRGAP2* expression ratio was similar among osteoblasts, primary osteosarcoma samples, and metastatic osteosarcoma samples (Fig. 4c). The level of *ENAH* expression, however, was significantly reduced in the metastatic osteosarcoma samples compared the osteoblasts (p = 0.0002) and primary osteosarcoma samples (p = 0.004). (Fig. 4d). Like SRGAP2, ENAH is an actin-associated protein involved in cytoskeleton remodeling, including lamellipodia and filopodia dynamics. These data demonstrate that *SRGAP2* and other genes in the Slit-Robo pathway may play a role in tumor formation in a subset of human osteosarcoma.

**Figure 5:**
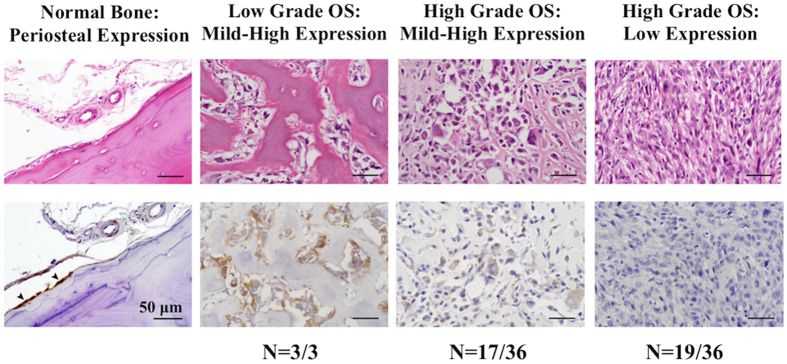
SRGAP2 protein expression is reduced or absent in half of high-grade human osteosarcoma tissue microarray samples. H&E staining (upper figures) and immunohistochemistry (IHC) (lower figures) were performed on normal bone and primary osteosarcoma samples purchased from Biomax. SRGAP2 immunolabelling is brown (DAB). SRGAP2 had mild-high expression in the periosteum of normal bone (arrows) and in low-grade osteosarcoma samples. SRGAP2 expression was minimal or not present in over half of high-grade, stage II osteosarcoma samples (n = 19/36). Low expression: no or minimal immunoreactivity. Mild-high expression: mild, moderated, or marked immunoreactivity. Scale bar: 50 μm.

Immunohistochemistry (IHC) and H&E staining was also performed on normal bone and primary osteosarcoma samples from humans (US Biomax, Rockville) (Fig. 5). The protein fragment used to raise the polyclonal antibody has regions of homology with SRGAP1. We evaluated the mRNA expression levels of SRGAP1 in the 12 juvenile osteosarcoma samples and osteoblast controls. While SRGAP1 is expressed in all of the samples, expression of SRGAP2 is on average 10 fold greater than SRGAP1. Therefore the amount of signal attributed to SRGAP1 is predicted to be minimal. Mild to high immuoreactivity to anti-SRGAP2 antibody was observed in the periosteum of normal bone and in low-grade osteosarcoma samples. In high-grade, stage II osteosarcoma samples, expression of SRGAP2 was substantially reduced or absent in over half of the samples (n = 19/36). Patient characteristics and scoring for each sample are listed in Supplementary Table S4.

### The Slit-Robo pathway is altered in Sleeping Beauty derived osteosarcoma

Given the increased expression of *ENAH* in human osteosarcoma samples, transposon insertion profiles for *Enah* were evaluated from the *Sleeping Beauty* derived tumors. In addition, transposon insertion profiles for *Slit2, Slit3*, and *Robo1* were evaluated given their direct upstream role in *SRGAP2* function. Insertions were found in primary tumors from animals with and without a *Trp53* deficient background in addition to transposon mutagenesis (n = 119) and in metastatic nodules (n = 19 animals; all metastatic nodules from one animal were grouped). Seven primary tumors (6%) and metastatic nodules from 4 animals (21%) had insertions in *Slit2*, 4 primary tumors (3%) and a metastatic nodule from one animal (5%) had insertions within *Slit3*, 3 primary tumors (3%) had insertions within *Robo1*, and 3 primary tumors (3%) and a metastatic nodule from 1 animal (5%) had insertions within *Enah* (Supplementary Fig. 2). In total, 28 primary tumors (24%) and metastatic nodules from 10 animals (53%) had a transposon insertion in at least one of the genes involved in the Slit-Robo pathway that were evaluated. *SRGAP2C* could not be evaluated by the *Sleeping Beauty* screen because it is only found in the human genome.

## Discussion

The nearly identical sequences shared between the ancestral and duplicated *SRGAP2* genes make it experimentally challenging to study *SRGAP2* in human cells. The murine genome has the advantage of containing only the ancestral *Srgap2* gene, which maintains a 98% amino acid sequence similarity with the human *SRGAP2* gene^10^. The data presented here support our hypothesis that SRGAP2 may have a role as a suppressor of metastases in osteosarcoma. Overexpression of *SRGAP2* reduced *in vitro* migration rates in both K12 and K7M2 cell lines. Knockout of *Srgap2* increased the *in vitro* migration rate of the less aggressive K12 cell line but not K7M2. It is possible the more aggressive K7M2 cell line already gained migration potential from another part of the Slit-Robo pathway or a parallel pathway when it was originally created. Although overexpression of *SRGAP2* also decreased the anchorage independent growth capacity of the K7M2 cell line, the absolute change may not be physiologically significant. Given the changes observed in *Srgap2* expression in a subset of primary tumors, it is possible that SRGAP2 also plays a role in primary tumor growth and later in metastasis. Unexpectedly, knockout of *Srgap2* slowed the proliferation of the K12 and K7M2 cell lines. This may be due to its role in F-actin dynamics. However, even with its affects on proliferation, *Srgap2* KO cell lines performed similarly to controls in the longer anchorage-independent growth assay.

SRGAP2 primarily functions in membrane dynamics, inducing filopodia formation through homodimerization^9^,^11^,^12^ and activating the GTPase of the F-actin modulating Rho-GAP protein Rac1^9^,^14^. Other modulators of Rac1 have also been implicated in osteosarcoma metastasis. Micro-RNA (miR)-142 has been shown to regulate Rac1 expression in osteosarcoma cell lines by targeting the 3’-untranslated region of the *SRGAP2* transcript and blocking translation of the protein^24^. Expression of miR-142 or direct silencing of Rac1 inhibited cellular invasion as well as cellular proliferation. In this work, the overexpression of *SRGAP2* also slowed cellular migration in murine osteosarcoma cell lines and the knockout of *Srgap2* increased cellular migration in the K12 cell line. However, unlike what has been observed in other studies of SRGAP2, knockout of *Srgap2* slowed cellular proliferation in osteosarcoma cell lines^16^,^24^.

In the *Sleeping Beauty* osteosarcoma mutagenesis screen, an enrichment of genes involved in axon guidance was found in pathway analysis of genes with high rates of transposon insertion^8^. SRGAP2 and SEMA4D have similar, but opposite effects on actin dynamics^8^,^10^,^25^. Unlike *SRGAP2*, the *SEMA4D* and *SEMA6D* genes had increased expression in mouse osteosarcoma tumors from the *Sleeping Beauty* screen and human osteosarcoma tissue samples^8^. Additionally, overexpression of *SEMA4D* or *SEMA6D* increased proliferation, colony formation in soft agar, and xenograft formation in OS cell lines^8^. These findings agree with the different effects of SRGAP2 and SEMA4D/SEMA6D on actin dynamics and further support the idea that genes involved in axon guidance have a role in metastatic osteosarcoma.

**Figure 6:**
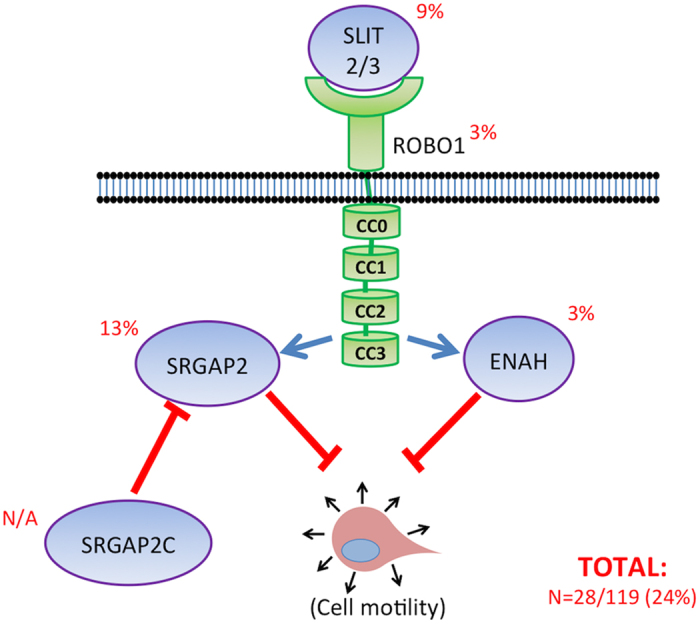
Effects on cell motility by SRGAP2, SRGAP2C, and ENAH in the Slit-Robo pathway. The diagram depicts the effects of SRGAP2C, SRGAP2, and ENAH that have been reported on cell motility in the Slit-Robo pathway. ENAH and SRGAP2 receive signaling from SLIT/ROBO that results in the suppression of cell motility. SRGAP2C inhibits the function of SRGAP2. The percent of *Sleeping Beauty* induced primary tumors with a transposon insertion in a given gene are listed next to its protein product in the diagram. Insertions were found in animals with and without a *Trp53* deficient background in addition to transposon mutagenesis (n = 28/119). Four primary tumors had a transposon insertion in more than one gene.

In this study, an increased *SRGAP2C*: *SRGAP2* transcript ratio or decreased expression of *ENAH* was found in all juvenile metastatic lesions (n = 7) compared to osteoblasts and primary tumor samples (n = 5). *ENAH-001* was the only detectable *ENAH* isoform in the St. Jude osteosarcoma^7^ and osteoblast control^8^ samples. The functions of SRGAP2, SRGAP2C, and ENAH are mediated by Slit-Robo signaling, but with different effects on cell motility. SRGAP2 and ENAH block cellular migration, whereas SRGAP2C promotes cellular migration through the inhibition of SRGAP2 (Fig. 6). Expression of SRGAP2C has been shown to increase motility in neuronal cells^10^ while decreasing ENAH-001 (Ensembl isoform designation) expression has been shown to increase motility in fibroblasts^26^,^27^. Transposon insertions were found in *Srgap2, Enah, Slit2, Slit3*, or *Robo1* in 24 percent of primary tumors from mice with *Sleeping Beauty* derived osteosarcoma, further implicating the role of the Slit-Robo pathway in osteosarcoma.

The migration results from the murine cell lines generally support the hypothesis that SRGAP2 acts as a suppressor of migration. This study also found that *SRGAP2, SRGAP2C*, and other genes in the Slit-Robo pathway have altered transcript levels in a subset of mouse and human osteosarcoma, and SRGAP2 protein expression is reduced or absent in half of primary tumor samples. SRGAP2 and other axon guidance proteins likely play a role in osteosarcoma metastasis, with loss of SRGAP2 contributing to a more aggressive phenotype. Further study of the axon guidance pathways may reveal new opportunities for osteosarcoma treatments.

## Methods

### Transposon integration site and RNA sequencing analysis of Sleeping Beauty derived osteosarcoma

The Integrative Genomics Viewer (IGV)^28^,^29^ was used to analyze the *Srgap2* insertion sites previously identified by Illumina sequencing in *Sleeping Beauty* derived mouse osteosarcoma tumors and metastatic nodules^8^. IGV was also used to analyze RNA sequencing data from 105 of the 119 *Sleeping Beauty* derived primary tumors. The samples were mapped using a modified genome containing a *Sleeping Beauty* chromosome with the T2/Onc sequence^21^.

### Creation and maintenance of cell lines

K7M2 and K12 cell lines were a kind gift from Chand Khanna and cultured in DMEM supplemented with 10% fetal bovine serum and 1% penicillin/streptomycin. *Srgap2* knockout was achieved using the CRISPR/Cas9 system (Addgene, Cambridge). A guide RNA (gRNA) was created to target exon 6 in murine cell lines (gcattgaggagaagcatgtc). A previously described method of co-transposition was used to enhance screening for knockout clones^30^. Briefly, cells were transfected with 2 μg Cas9 nuclease, 2 μg gRNA, 500 ng CMV-PB7 *PiggyBac* transposase, and 500 ng CAGG-Luciferase-IRES-GFP-PGK-Puro *PiggyBac* transposon for resistance to puromycin. Electroporation was performed on one million cells in 100 μL of PBS using the NEON transfection system (Thermo Fisher, Waltham), following the manufacturer’s protocol. After two days of incubation, cells were plated into 96 well plates at densities ranging from 50–1,000 cells per well. Puromycin was added at a concentration of 2 μg/mL of culture media, and wells later growing single colonies were selected for knockout analysis.

Conditional *PiggyBac* vectors were utilized for *SRGAP2* overexpression. *SRGAP2* cDNA with the human sequence was transferred into the previously described PB-TRE-DEST1-EF1A-rtTA-IRES-Puro by a standard LR Clonase reaction (Thermo Fisher), following manufacturer’s instruction^18^,^30^. Luciferase control cell lines were created using the same CAGG-Luciferase-IRES-GFP-PGK-Puro *PiggyBac* transposon for resistance to puromycin used in the knockout cell lines. Electroporation was performed on one million cells in 100 μL PBS with 2 μg *PiggyBac* vector containing the transgenes for *SRGAP2* and puromycin resistance or luciferase and puromycin resistance and 2 μg CMV-PB7 *PiggyBac* transposase. Following two days of incubation, cells were treated with 2 μg puromycin/mL of media to obtain a polyclonal population with an integrated transgene. *SRGAP2* cDNA expression was induced through the addition of 2.5 μg doxycycline/mL of media 48 hours prior to the initiation of experiments.

### Analysis of knockout clones

A 350 base pair region spanning the CRISPR gRNA target sequence was PCR amplified and sequenced to assess for genomic mutations (for: ggccctgctgagttttgtta, rev: atcaatcttgccttccaagc). No commercial antibodies were found to bind murine Srgap2 for Western blot analysis. Quantitative RT-PCR (qPCR) was, therefore, used to assess knockout of *Srgap2* in K12 and K7M2 cell lines. Finally, to test for heterogeneity among the different copies of the gene within cells, the TOPO TA Cloning Kit for Sequencing (Thermo Fisher) was used to sequence individual copies of *Srgap2*. PCR products from each cell line were inserted into the TOPO vectors, and nine to ten colonies of competent bacteria for each knockout cell line were selected for sequencing.

### Quantitative RT-PCR

RNA was extracted from cell lines using the High Pure RNA Isolation Kit (Roche, Basel). 1 μg of extracted RNA was reverse transcribed into cDNA using the Transcriptor First Strand Synthesis kit (Roche). Quantitative RT-PCR was performed in triplicate using SYBR green mix (Qiagen, Hilden) on an ABI 7500 machine (Applied Bio Systems, Foster City). Data was analyzed using Microsoft Excel and graphed using the Prism software package. The following primer sequences were used: *SRGAP2* (for: aggaggaagcatggaggatt, rev: ttcatcatcgcttgtgtggt), *Gapdh* (for: gtgttcctacccccaatgtgt, rev: gagacaacctggtcctcagtgt). Data was graphed and analyzed using the Prism software package. Two-tailed, unpaired t-tests were used to determine statistical significance (p < 0.05).

### Western blot analysis

Protein was extracted from cultured cells in a NP-40 buffer (50 nM Tris HCL pH 7.6, 150 mM NaCl, 1% NP-40, 5 mM NaF, 1 m MEDTA; Thermo Fisher) containing a protease inhibitor (Roche) and phosphatase inhibitors (Sigma-Aldrich, St. Louis). Protein samples were run on 10% Bis-Tris gels (NuPage, Thermo Fisher) and transferred to PVDF membranes. The membranes were blocked in 5% nonfat dry milk for 1 hour and then incubated with primary antibody for 3 hours: SRGAP2 (1:1000, Abcam, Cambridge, #ab121977) and β-actin (1:2000, Abcam, #ab8227-50). Subsequently, membranes were incubated in goat anti-rabbit IgG-HRP conjugated secondary antibody (1:5000, Santa Cruz, Dallas, #sc-2004) for one hour. Blots were thoroughly washed and developed using the WesternBright Quantum detection kit (Advansta, Melano Park) and Licor Odyssey Image Studio.

### MTS proliferation assay

Two thousand cells were plated into 6 wells of a 96-well plate for each cell line, with three replicates per line, for a total of 18 wells. Cells were incubated for 24, 48, 72, and 96 hours, at which time 20 μL of a 1:20 mixture of phenazine methosulfate (PMS): 3-(4,5-dimethylthiazol-2-yl)-5(3-carboxymethonyphenol)-2-(4-sulfophenyl)-2H-tetrazolium (MTS) was added to each well. Cells were incubated for 4 hours and then absorbance at 490 nm and 650 nm was read using the BioTek SynergyMx fluorescence plate reader. Normalized absorbance was obtained by subtracting the reading at 650 nm from the reading at 490 nm and further subtracting the average reading of 6 media control wells. Data was graphed using the Prism software package. Due to multiple time points for each cell line, one-way analysis of variance (ANOVA) was conducted at each time point (24 hours, 48 hours, 72 hours, and 96 hours). Significance was set at p < 0.01 Unpaired student t-tests were used to compare specific time points (p < 0.05). Fold change for each time point was also calculated compared to 24 hours to evaluate changes in proliferation rates.

### Wound healing assay

Cells were incubated for 24 hours in silicone inserts with two wells separated by a width of 500 μm +/− 50 μm (ibidi, Martinsried) in 35 mm dishes. Cell seeding was determined by plating 25, 30, 35, 40, 45, and 50 × 10^3^ cells in insert wells. The lowest number of cells for which all transformed cell lines from each set were confluent at 24 hours was used for experiments: K12 (35,000), K7M2 (50,000). Doxycycline was added to cells 24 hours prior to placement in inserts; cells were exposed to doxycycline for 48 hours prior to imaging. After inserts were removed, cells were rinsed with PBS, and media with or without doxycycline was added to the dishes. For time lapse imaging, cell culture dishes were placed in a Bold Line top stage incubator (Okolab, Pozzuoli) at 37 °C and 5% CO_2_. Phase contrast images of the wounds were acquired every 15 minutes for 15 hours using a Nikon 10X (0.25NA Ph1 ADL) objective and a Nikon TiE stand with a Zyla 5.5 sCMOS camera (Andor Technology, Belfast). Large image function on NIS element and Nikon Perfect Focus system were used to acquire large images of the entire wound.

A custom-written image segmentation algorithm in MATLAB was used to measure wound closure rate. Cell edges were identified using the Canny edge detection algorithm in MATLAB. The identified edges were then dilated to bridge any gaps in the edge detection. Regions enclosed by the edge detection were filled and eroded back to their original size (Supplementary Fig. S2). Total unmasked area (gap area) was then measured and divided by the image length to calculate the gap width for each frame. Finally, the wound closure rate was determined by calculating the slope of the linear segment of a gap width vs. time curve (Supplementary Fig. S2). A minimum of 16 inserts was analyzed for each condition. Results were graphed using the Prism soft ware package, and statistical significance was determined with unpaired student t-tests (p < 0.05).

### Anchorage-independent growth assay

A layer of sea plaque agar (Lonza, Basel) at a concentration of 0.8% in culture media was placed into 6-well plates and allowed to solidify. A top layer was then placed into each well containing 10,000 cells in 0.48% agar in culture media and allowed to solidify. One mL of culture media was placed on top of the solidified agar with or without 2.5 μg doxycycline/mL of media. Plates were incubated for 2 weeks, with one mL of culture media added to every well after one week. After two weeks, the media was removed and cells were fixed in 10% buffered formalin (Thermo Fisher) containing 0.5% crystal violet for 2 hours. Each well was divided into four quadrants and photographed on a Leica S8 AP0 microscope. Colonies were quantified using ImageJ software and graphed using the Prism software package. Three wells were used for each condition, conducted in triplicate, giving 9 wells per condition and 36 quadrants for imaging. Two-tailed, unpaired t-tests with α = 0.05 were used to identify statistically significant differences in colony numbers (p < 0.05).

### Immunohistochemistry

Tissue microarrays were purchased from Biomax (Normal bone: BO244d, Osteosarcoma: OS804a). Slides containing 4 μm thick formalin-fixed, paraffin-embedded sections of tumor/control bone tissue were deparaffinized and rehydrated. Antigen retrieval was performed in a steamer using 1 mM Tris base EDTA buffer, pH 9.0. After endogenous peroxidase blocking, a protein block was applied. Immunohistochemistry (IHC) for SRGAP2 was performed using rabbit anti-SRGAP2 (Sigma-Aldrich HPA-028191) primary antibody on a Dako Autostainer. Detection was achieved using the Dako Envision rabbit detection system with diaminobenzidine (Dako, Glostrup) as the chromogen. Sections were counterstained with Mayer’s Hematoxylin (Dako). Xenograft tumors confirmed by western blot to express SRGAP2 were used as positive control.

Tissue sections were imaged on a Nikon E800M microscope at 40X magnification using a Nikon DSRi2 camera and Nikon Elements D Version 4 software. Each section was imaged using the same white balance and shading correction settings. Whole image sharpness was uniformly adjusted with Photoshop Elements version 11 software.

## Additional Information

**How to cite this article**: Marko, T. A. *et al*. Slit-Robo GTPase-Activating Protein 2 as a metastasis suppressor in osteosarcoma. *Sci. Rep.* **6**, 39059; doi: 10.1038/srep39059 (2016).

**Publisher's note:** Springer Nature remains neutral with regard to jurisdictional claims in published maps and institutional affiliations.

## Supplementary Material

Supplementary Information
