# Supplementary material for: Slit-Robo GTPase-Activating Protein 2 as a metastasis suppressor in osteosarcoma
Source: Sci Rep. 2016 Dec 14;6:39059. doi: 10.1038/srep39059 (PMC5155223; doi:10.1038/srep39059)
Supplement: Supplementary Information [file srep39059-s1.pdf]

Slit-Robo GTPase-Activating Protein 2 as a metastasis suppressor in osteosarcoma

Tracy A. Marko[1], Ghaidan A. Shamsan[2], Elizabeth N. Edwards[1], Paige E. Hazelton[1], Susan K. Rathe[1], Ingrid Cornax[1,3], Paula R. Overn[1,3], Jyotika Varshney[1], Brandon J. Diessner[4], Branden S. Moriarity[1,4,5], M. Gerard O'Sullivan[1,3,6], David J. Odde[2], David A. Largaespada*[1,4]

University of Minnesota: (1) Masonic Cancer Center (2) Department of Biomedical Engineering (3) Comparative Pathology Shared Resource (4) Department of Pediatrics (5) Center for Genome Engineering (6) College of Veterinary Medicine, Department of Veterinary Population Medicine

.

Supplementary Figure S1. ***Srgap2*** **sequence reads around guide RNA target region in exon 6 of murine cell lines.**

K12/ K7M2 WT	g	t	c	c	g	c	a	t	t	g	a	g	g	a	g	a	a	g	c	a	t		g	t	**c**	c	g	g	a	g	g	a	g	**Mutation**	**N**
K7M2 KO	g	t	c	c	g	c	a	t	t	g	a	g	g	a	g	a	a	g	c	a	t	**T**	g	t	**c**	c	g	g	a	g	g	a	g	1 BP Insertion	3
	g	t	c	c	g	c	a	t	t	g	a	g	g	a	g	a	a	g	c	a	**-**		g	t	**c**	c	g	g	a	g	g	a	g	1 BP Deletion	2
	g	t	c	c	g	**-**	**-**	**-**	**-**	**-**	**-**	**-**	**-**	**-**	**-**	**-**	**-**	**-**	**-**	**-**	**-**		**-**	**-**	**-**	**-**	**-**	g	a	g	g	a	g	21 BP Deletion	4
K12 KO	g	t	c	c	g	c	a	t	t	g	a	g	g	a	g	a	a	g	c	a	t	**T/A**		t	**c**	c	g	g	a	g	g	a	g	SNP	3
	g	t	c	c	g	c	a	t	t	g	a	g	g	a	g	a	**-**	**-**	**-**	**-**	**-**		g	t	**c**	c	g	g	a	g	g	a	g	5 BP Deletion	4
	g	t	c	c	g	c	a	t	t	g	a	g	g	a	g	a	a	g	c	a	t	**A**	g	t	**c**	c	g	g	a	g	g	a	g	1 BP Insertion	2
	g	t	c	c	g	c	a	t	t	g	a	g	g	a	g	a	a	**T**	c	**C**	t		g	**A**	**c**	**G**	**A**	g	a	T	**C**	a	g	SNP	1

Genomic sequencing was performed on the murine knockout cell lines. The guide RNA target sequence is highlighted in gray. Insertions and deletions, which all occurred within or immediately following the target sequence, are highlighted in dark gray. The wild type sequence is shown above the knockout sequences. WT: Wild Type. N: Number of reads of a particular sequence. BP: Base pair. SNP: Single nucleotide polymorphisms- single base pair substitutions.

Supplementary Figure S2. ***Sleeping Beauty*** **transposon insertion sites within** ***Enah, Slit2, Slit3,*** **and** ***Robo1***

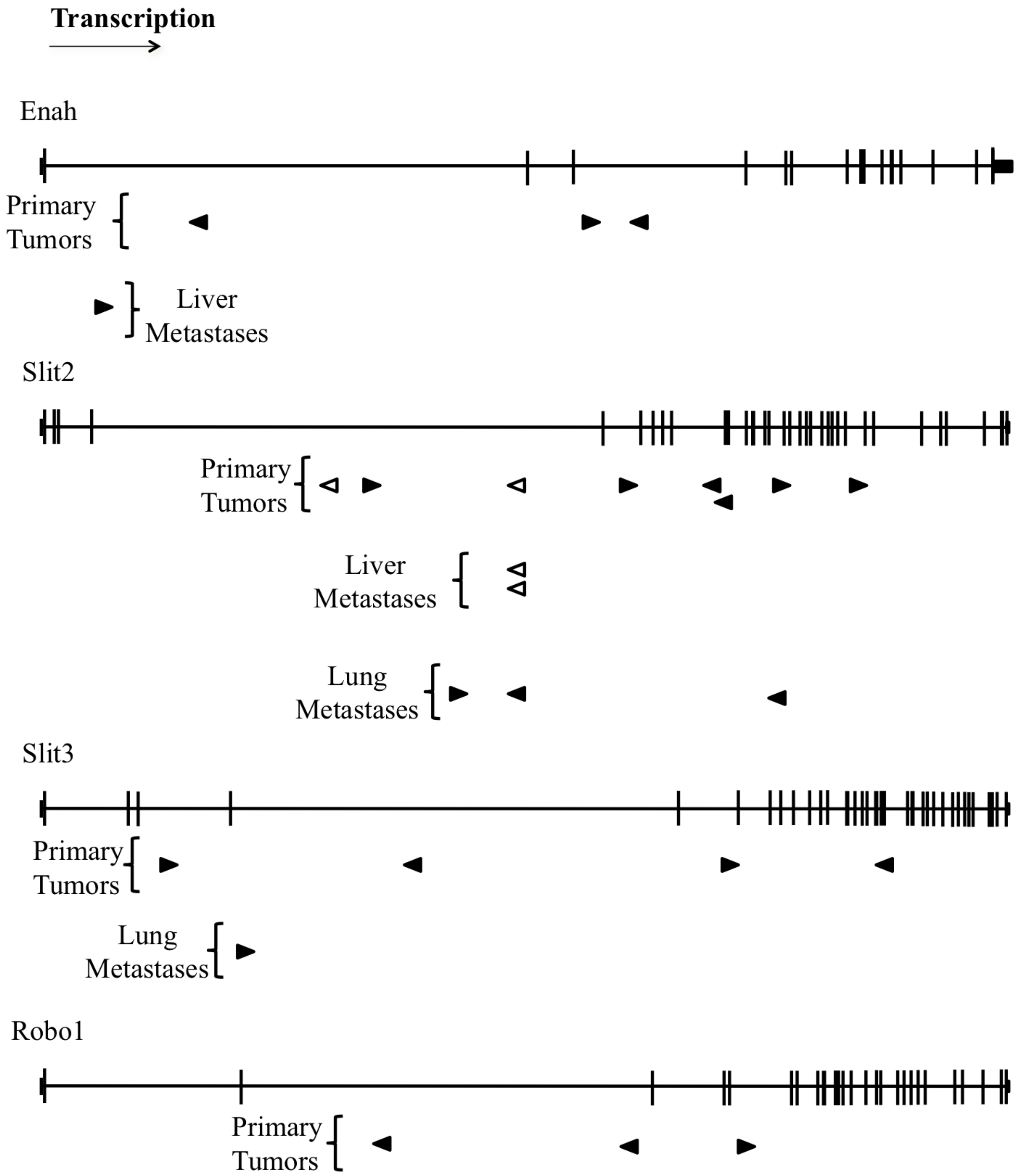

Arrows denote transposon insertion sites, which point in the direction of the promoter. One animal had multiple insertions within *Slit2*, denoted by the open arrows. The insertions represented by solid arrows within each gene are from different animals.

Supplementary Figure S3. Illustration of wound closure assay analysis

a

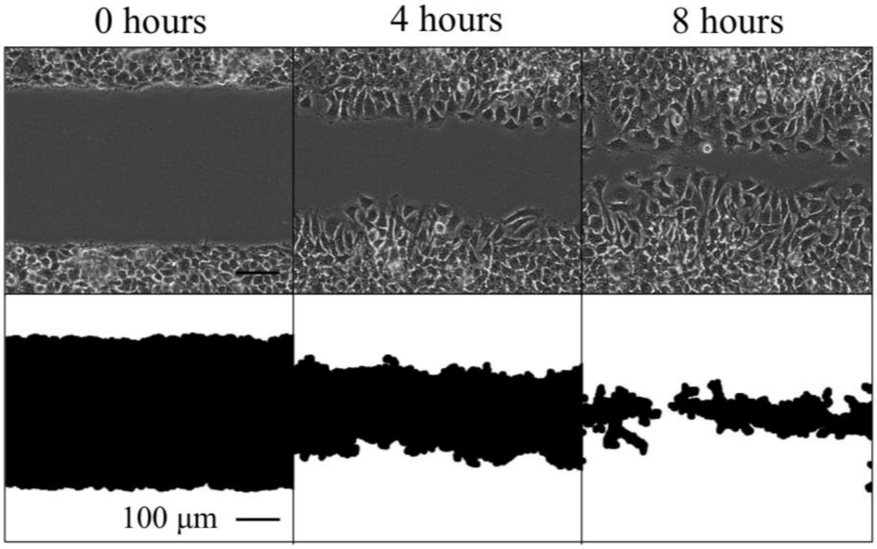

b

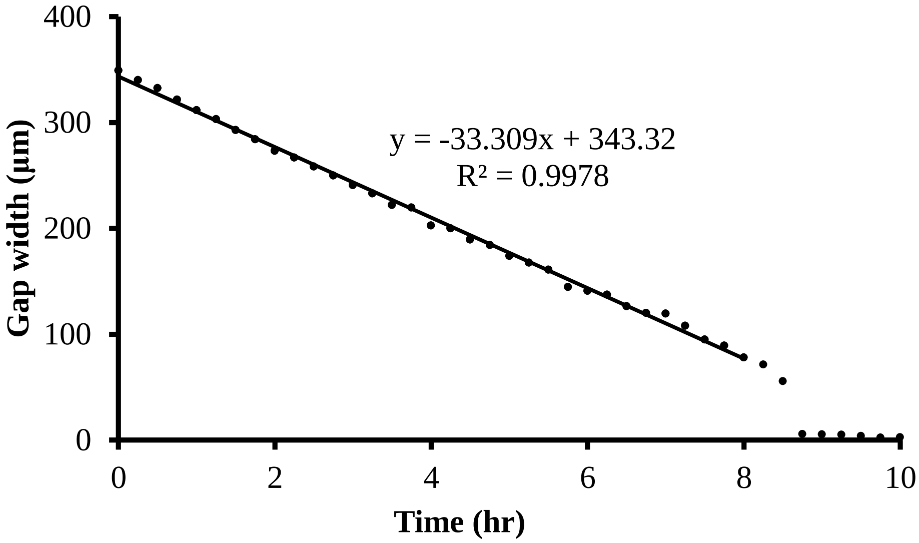

(a) A montage of phase contrast images of wound closure (K12 Luci - Dox) and the corresponding segmented images generated in MATLAB. (b) Gap width vs. time plot corresponding to the wound in (a) illustrating the extraction of closure rate by fitting the linear segment of the curve to a line equation where the slope is the closure rate. Scale bar 100 μm.

Supplementary Table S1: ***Sleeping Beauty*** **mutagenesis screens identify** ***Srgap2*** **as a candidate tumor driver gene**

**Study**	**Predicted effect on gene function**	**Cancer Type**	**Percent of Animals with Insertions in Primary Tumor**
Moriarity et al. 2015	Disruption	Osteosarcoma	13.5% (n = 13/96)
Giotopoulos et al. 2015	Not evaluated	Chromic myeloid leukemia	30% (n = 9/30)
Rahrmann et al. 2013	Disruption	Peripheral nerve sheath tumor	16-18% (n= 17-19/106)
Genovesi et al. 2013	Not evaluated	Medulloblastoma	14% (n = 12/85)

Supplementary Table S2. IGV analysis results on the impact of SB insertions into *Srgap2* in SB-induced mouse osteosarcomas

Sample	Search for SB containing transcripts in *Srgap2* using IGV
OM067*	*Srgap2* reads paired to SB reads found in exons 4 and 5 pointing toward intron 5. Sections of intron 5 spliced into transcript. 18 of the 106 reads (17%) at the end of exon 4 are paired to SB.
OM218*	2 *Srgap2* reads related to SB read found in exon 1 pointing toward intron 1. No normal reads spanning intron 1.
OM272*	Nearly half of the *Srgap2* reads found in exons 3 and 4 are paired to SB and are pointing to intron 4. 62 normal reads spanning intron 2 while only 7 normal reads spanning intron 4 (89% drop in expression).
OM343*	6 *Srgap2* reads related to SB read found in exon 3 pointing toward intron 4. 27% drop in reads spanning intron 3 compared to intron 2.
OM347	1 *Srgap2* read related to SB read found in exon 4 pointing toward intron 3. No apparent drop in expression.
OM374B*	Nearly half of the *Srgap2* reads found in exon 3 are paired to SB and pointing toward intron 3. Drop in expression starting in exon 4. 65% drop in reads spanning intron 3 compared to reads spanning intron 2.
OM380	*Srgap2* reads paired to SB read found in exons 10-13 pointing toward intron 14. No apparent drop in normal expression.
OM411C*	1 *Srgap2* read related to SB read found in exon 4 pointing toward intron 5. No normal reads spanning intron 4.
OM445	2 *Srgap2* reads related to SB read found in exon 7 pointing toward intron 7. No apparent drop in expression.
OM520*	*Srgap2* reads related to SB read found in exon 7 pointing toward intron 7. 31% drop in normal reads spanning intron 7 compared to reads spanning intron 6.

*Samples with reduction in normal SRGAP2 expression by RNA sequencing.

Supplementary Table S3. ANOVA analysis of MTS proliferation assay

	K12 Cell lines: Mean (SEM)					
	Lucif - Dox	Lucif + Dox	OE - Dox	OE + Dox	KO	P-value
24 hours	0.24 (0.002)	0.23 (0.001)	0.26 (0.002)	0.26 (0.003)	0.35 (0.002)	5.40 E -15
48 hours	0.51 (0.004)	0.47 (0.003)	0.44 (0.003)	0.51 (0.004)	0.57 (0.005)	1.79 E-06
72 hours	0.77 (0.008)	0.69 (0.006)	0.53 (0.005)	0.62 (0.004)	0.75 (0.006)	5.27 E-10
96 hours	1.21 (0.006)	1.33 (0.007)	0.90 (0.008)	1.14 (0.005)	0.93 (0.006)	< 2.2 E-16
Fold change 96 v 24	5.04	5.78	3.46	4.38	2.66	
Fold change 72 v 24	3.21	3.00	2.04	2.38	2.14	
Fold change 48 v 24	2.13	2.04	1.69	1.96	1.63	

	K7M2 Cell lines: Mean (SEM)					
	Parent - Dox	Parent + Dox	OE - Dox	OE + Dox	KO	P-value
24 hours	0.69 (0.004)	0.68 (0.005)	0.47 (0.001)	0.45 (0.002)	0.58 (0.004)	<2.2 E-16
48 hours	1.11 (0.004)	1.03 (0.004)	0.81 (0.004)	0.76 (0.003)	0.7 (0.004)	<2.2 E-16
72 hours	1.28 (0.012)	1.32 (0.004)	1.12 (0.004)	1.14 (0.004)	0.85 (0.01)	<2.2 E-16
96 hours	1.62 (0.008)	1.57 (0.007)	1.56 (0.008)	1.3 (0.007)	1.24 (0.006)	6.03 E-16
Fold change 96 v 24	2.35	2.31	3.32	2.89	2.14	
Fold change 72 v 24	1.86	1.94	2.38	2.53	1.47	
Fold change 48 v 24	1.61	1.51	1.72	1.69	1.21	

Supplementary Table S4. Patient characteristics and IHC score for osteosarcoma TMA

Position on Slide	Sex	Age	Location of tumor	Size of tumor	Grade	srGAP2 score
A1, A2	M	47	Right femur	>8 cm	High	1
A3, A4	M	38	Left tibia	>8 cm	High	1
A5, A6	M	32	Right femur	>8 cm	High	3
A7, A8	M	42	Right femur	>8 cm	High	0
A9, A10	M	11	Left femur	>8 cm	High	1
B1, B2	F	16	Left femur	>8 cm	High	0
B3, B4	M	38	Left femur	>8 cm	High	3
B5, B6	M	41	Rib	≤8 cm	High	2
B7, B8	F	32	Right femur	>8 cm	High	0
B9, B10	F	15	Left femur	>8 cm	High	0
C1, C2	M	51	Left femur	>8 cm	High	1
C3, C4	F	12	Right femur	≤8 cm	High	1
C5, C6	F	14	Right femur	>8 cm	High	3
C7, C8	M	13	Left tibia	≤8 cm	High	2
C9, C10	F	44	Right scapula	≤8 cm	High	2
D1, D2	F	17	Right femur	>8 cm	Low	NE*
D3, D4	F	47	Left femur	>8 cm	High	0
D5, D6	F	14	Right femur	>8 cm	High	3
D7, D8	M	41	Right tibia	>8 cm	Low	3
D9, D10	F	32	Right humerus	>8 cm	High	3
E1, E2	F	14	Left tibia	>8 cm	High	0
E3, E4	M	19	Left femur	>8 cm	High	2
E5, E6	M	16	Left femur	>8 cm	High	0
E7, E8	M	18	Right humerus	>8 cm	High	2
E9, E10	M	23	Left femur	>8 cm	High	1
F1, F2	M	60	Right tibia	>8 cm	High	2
F3, F4	M	31	Left humerus	>8 cm	High	1
F5, F6	M	37	Left femur	≤8 cm	High	3
F7, F8	M	30	Right femur	>8 cm	High	3
F9, F10	F	32	Left femur	>8 cm	High	0
G1, G2	M	29	Left femur	≤8 cm	High	3
G3, G4	M	64	Left femur	>8 cm	High	2
G5, G6	M	35	Left femur	>8 cm	High	0
G7, G8	M	21	Left femur	>8 cm	High	0
G9, G10	M	51	Right fibula	>8 cm	High	0
H1, H2	M	32	Rib	≤8 cm	High	0
H3, H4	F	38	Right scapula	≤8 cm	Low	2
H5, H6	M	43	Right femur	≤8 cm	Low	3
H7, H8	M	44	Right femur	>8 cm	High	4
H9, H10	M	17	Right femur	>8 cm	High	4
Control tissue	N/A	N/A	N/A	N/A	N/A	0

*NE: Not evaluated due to poor tissue quality.

Supplementary Table S5. Gene expression levels (FPKMs) and *SRGAP2C*: *SRGAP2* ratio in genes involved in the Slit-Robo pathway in St. Jude juvenile osteosarcoma samples

	Control Samples*			Primary Tumor Samples					Metastatic Lesion Samples						
**Gene**	**OB01**	**OB02**	**OB03**	**OS03**	**OS05**	**OS09**	**OS12**	**OS20**	**OS04**	**OS07**	**OS08**	**OS11**	**OS13**	**OS16**	**OS29**
ABL1	38.88	41.05	38.66	21.96	23.44	16.44	9.22	20.63	24.70	19.37	17.79	6.84	20.43	26.10	28.28
ARPC2	288.31	327.01	301.98	209.59	207.08	237.18	360.16	158.35	241.28	278.43	168.52	297.06	270.48	186.08	175.49
ARPC3	185.22	209.46	197.35	176.72	120.92	123.30	155.72	154.29	211.29	193.64	301.07	264.35	166.62	225.33	174.58
CDC42	93.07	98.88	99.69	117.72	185.33	147.65	161.27	127.30	178.32	158.08	223.49	96.89	222.03	160.23	193.50
CLASP1	8.81	7.71	7.75	7.78	10.65	13.00	17.95	9.69	11.17	6.00	5.66	2.90	10.10	6.37	9.72
CLASP2	6.03	5.96	5.52	7.11	5.31	5.84	4.81	19.34	6.68	6.52	1.56	2.52	6.94	6.25	5.44
CXCL12	97.25	98.79	92.24	26.87	12.76	1.52	9.87	3.71	89.70	3.89	3.12	36.04	28.90	17.70	47.34
CXCR4	0.00	0.00	0.05	45.93	55.96	33.87	34.23	13.85	49.89	57.48	12.85	33.67	30.88	49.63	21.12
DCC	0.01	0.02	0.01	0.00	0.01	0.02	0.37	0.07	0.10	0.05	0.00	0.01	0.25	0.20	0.04
ENAH	23.29	24.02	22.13	18.48	27.68	23.16	17.03	13.62	9.00	9.22	9.63	6.21	11.50	13.26	15.79
RAC1	104.40	109.08	103.18	130.71	170.19	203.79	117.40	128.00	240.59	318.97	128.36	151.84	127.16	131.60	100.77
RHOA	385.45	413.34	389.46	252.62	207.35	183.37	177.21	184.90	428.92	228.32	147.73	258.38	278.03	368.29	309.53
ROBO1	12.51	11.94	11.25	15.67	6.59	16.50	21.35	17.39	8.47	9.13	3.88	1.72	10.72	15.31	22.71
ROBO2	0.02	0.02	0.02	1.91	16.34	4.89	1.71	9.32	0.18	6.32	0.82	1.37	5.88	2.43	0.39
ROBO3	5.27	4.01	3.68	2.46	1.57	1.70	0.79	1.86	1.15	1.92	11.14	2.51	1.10	2.10	4.75
SLIT1	0.03	0.03	0.03	0.12	0.06	0.06	0.10	0.07	0.09	0.12	0.04	0.06	0.06	0.07	0.08
SLIT2	17.15	18.35	16.69	42.90	49.92	1.99	12.23	11.94	23.77	3.83	3.24	2.69	7.26	9.98	16.24
SLIT3	28.86	31.08	27.84	34.62	39.50	41.92	7.54	54.61	66.37	22.98	11.37	4.30	36.66	29.70	21.93
WASL	11.53	12.43	11.53	13.20	23.64	13.86	11.33	14.72	19.33	9.10	7.78	6.68	17.79	20.24	31.70
SRGAP2C:2 ratio	0.78	0.77	0.75	0.64	1.14	1.00	0.60	1.20	0.39	0.69	0.36	0.88	0.35	1.49	1.46

*Control samples are 3 samples from the same patient.

2+ fold decrease from osteoblast control samples (1.5+ in the *SRGAP2C:2* ratio)

2+ fold increase from osteoblast control samples (1.5+ in the *SRGAP2C:2* ratio)

Supplementary Table S6. Naming convention for St. Jude juvenile osteosarcoma samples

Sample	Name of left read fastq files	Name of right read fastq files
OS03	SJOS001103_D1-TB-08-1096L	SJOS001103_D1-TB-08-1096R
OS04	SJOS001104_M1-TB-11-65000L	SJOS001104_M1-TB-11-65000R
OS05	SJOS001105_D1-TB-08-23890L	SJOS001105_D1-TB-08-23890R
OS07	SJOS001107_M1-TB-09-29300L	SJOS001107_M1-TB-09-29300R
OS08	SJOS001108_M1-TB-09-55200L	SJOS001108_M1-TB-09-55200R
OS09	SJOS001109_D1-TB-12-04920L	SJOS001109_D1-TB-12-04920R
OS11	SJOS001111_M1-TB-97-01070L	SJOS001111_M1-TB-97-01070R
OS12	SJOS001112_D1-TB-09-04800L	SJOS001112_D1-TB-09-04800R
OS13	SJOS001113_M1-TB-09-30590L	SJOS001113_M1-TB-09-30590R
OS16	SJOS001116_M1-TB-09-18980L	SJOS001116_M1-TB-09-18980R
OS20	SJOS001120_D1-TB-09-52820L	SJOS001120_D1-TB-09-52820R
OS29	SJOS001129_M2-TB-11-37400L	SJOS001129_M2-TB-11-37400R
